# Gene-gene and gene-environment interactions of *CYP19A1, ESR1, IL6, IL6R, IL1β, RANK, and RANKL* variants in relation to osteoporosis and hip fracture risk in Mexican women

**DOI:** 10.3389/fragi.2026.1769306

**Published:** 2026-03-03

**Authors:** Antonio Miranda-Duarte, Valeria Ponce de León-Suárez, Alberto Hidalgo-Bravo, Rafael Velázquez-Cruz, Esperanza Ramírez-Pérez, O. Celeste Martínez-Ramírez, Clementina Castro-Hernández, Blanca Barredo-Prieto, Leonora Casas-Avila

**Affiliations:** 1 Medicina Genómica, Instituto Nacional de Rehabilitación, Mexico City, Mexico; 2 Laboratorio de Genómica del Metabolismo Óseo, Instituto Nacional de Medicina Genómica (INMEGEN), Mexico City, Mexico; 3 Facultad de Nutrición, Universidad Autónoma del Estado de Morelos, Cuernavaca, Morelos, Mexico; 4 Medicina Genómica y Toxicología Ambiental, Instituto de Investigaciones Biomédicas, Universidad Nacional Autónoma de México, Mexico City, Mexico

**Keywords:** gene-environment interaction, gene-gene interaction, hip fracture, osteoporosis, single nucleotide variants

## Abstract

**Background:**

Osteoporosis is a complex disease influenced by genetic variants, environmental factors, and comorbidities. While individual single-nucleotide variants (SNVs) have been associated with disease risk, limited data are available on how gene–gene and gene–environment interactions contribute to osteoporosis and fracture susceptibility in Mexican women.

**Method:**

In this case–control study, we evaluated the association of SNVs in estrogen receptor alpha (*ESR1*), aromatase (*CYP19A1*), interleukin 6 (*IL6*) and its receptor (*IL6R*), interleukin 1 beta (*IL1β*), Receptor activator of nuclear factor κ B (*RANK*) and its ligand (*RANKL*) genes, with the risk of osteoporosis and hip fracture, as well as their gene-gene and gene-environment interactions, in 609 Mexican women (169 with osteoporosis, 205 with hip fracture, and 235 controls), by real time PCR with TaqMan probes. In addition, multifactor dimensionality reduction (MDR) software was used to detect gene–gene interactions and gene-environment interactions.

**Results:**

The GA and AA genotypes of *RANK* rs3018362 were significantly associated with increased osteoporosis risk (OR = 2.08 [1.08–3.98] and 2.76 [1.21–6.30], respectively) while the CC genotype of ESR1 rs2234693 was associated with reduced risk (OR = 0.28 [0.11–0.69]). For hip fracture, *ESR1* rs2234693 (CC genotype) was protective (OR = 0.30 [0.12–0.75]), whereas *RANK* rs3018362 (AA genotype) increased risk (OR = 2.4 [1.01–6.06]). A significant gene–gene interaction between *ESR1* (rs2228480) and *RANK* (rs3018362) increased osteoporosis risk (OR = 2.1 [1.4–3.2], CVC = 10/10), and a gene–gene model involving *ESR1*, *IL6R*, *IL1β*, and *RANKL* was identified for hip fracture (CVC = 8/10). In osteoporosis, a gene–environment interaction was observed between *CYP19A1* SNVs (rs700518, rs1062033, rs4775936, rs767199), *IL1β* rs16944, and 10-year probability of major fracture (CVC = 10/10).

**Conclusion:**

Our findings suggest that *RANK* and *ESR1* variants are independently and interactively associated with osteoporosis and fracture risk, and that gene–gene and gene–environment interactions play a critical role in disease susceptibility among Mexican women.

## 1 Introduction

Osteoporosis (OP) is a complex skeletal disease characterized by a generalized reduction in bone mineral density (BMD) and microarchitectural deterioration of bone tissue. It is clinically important due to its strong association with fragility fractures, particularly of the hip (HFx). As a multifactorial disease, it is well recognized that the interplay of genetic, environmental, and clinical factors significantly contributes to its development. The primary risk factors associated with OP include aging, low body mass index, prior fractures, and muscle weakness. In addition, numerous studies have demonstrated that genetic factors play an important role in OP susceptibility.

Among the various mechanisms involved in OP, decreased serum estrogen concentrations are a well-established contributor to disease pathogenesis. After menopause, the aromatization of androgenic precursors becomes the principal source of estrogen in women. Therefore, the estrogen receptor alpha (*ESR1*) gene and the aromatase (*CYP19A1*) gene, which encodes the enzyme responsible for the conversion of androgens to estrogens, are considered strong candidate genes for OP. Associations between single-nucleotide variants (SNVs) in *ESR1* and *CYP19A1* and osteoporosis have been extensively reported, although findings have varied across populations. For example, a meta-analysis found associations between *ESR1* SNVs and OP that differed by ethnicity (Zhu et al., 2018; Shu et al., 2020). Similarly, one haplotype in *CYP19A1*, formed by SNVs rs700518, rs767199, rs4775936, and rs1062033, which are highly frequent in the Spanish population, was associated with increased OP risk and low *CYP19A1* expression (Riancho et al., 2007). Individually, rs700518 and rs1062033 were associated with OP in a meta-analysis (Ma et al., 2020) and in Spanish women (Riancho et al., 2009), but not in Polish women (Kamiński et al., 2019).

Inflammatory cytokines also play an important role in OP. Interleukin 6 (IL6), interleukin 1 beta (*IL1β*), and their respective receptors exert significant effects on osteoclasts genesis and maturation, and bone resorption. Genetic variants in *IL6* and *IL6R* have been associated with OP risk. One of the most studied SNVs in *IL6* is rs1800795 (G174C), which has been described as a protective factor against osteoporosis in Caucasian and Asiatic populations (Ferrari et al., 2003; Chung et al., 2003). As part of a haplotype with other variants, rs1800795 was also associated with a favorable response to alendronate treatment in Mexican women (Villagómez Vega et al., 2023). Another SNV in IL6, rs1800796 (G572C), has been linked to BMD variations in Japanese women (Oishi et al., 2012) and a higher risk of vertebral compression fractures in Chinese women (Xiong et al., 2022). However, in Iranian patients, this SNV was not associated with BMD, but was linked to serum calcium levels (Eftekhari et al., 2018). The *IL1β* polymorphism rs1143634 has been associated with low BMD and OP risk in Taiwanese women (Chen et al., 2003) but not in Polish women (Czerny et al., 2010).

Disorders in bone metabolism are central to the pathogenesis of osteoporosis. The signaling system comprising the receptor activator of nuclear factor-κB (RANK), its ligand (RANKL), and osteoprotegerin (OPG) play a crucial role in coupling osteoblast and osteoclast activity to regulate bone remodeling. Conflicting results have been reported regarding the association of these genes with OP. The A allele of *RANK* rs3018362 has been associated with low BMD and an increased risk of fracture in European and Chinese populations (Paternoster et al., 2010; Liu et al., 2010). Conversely, in another study in Chinese women, the G allele of this SNV was associated with low BMD (Shang et al., 2013).

A range of lifestyle factors, including physical activity, smoking, alcohol consumption, and calcium and protein intake (Zhu and Prince, 2015), as well as the presence of comorbidities, may interact with genetic predispositions and influence OP risk. Most studies have focused on single-gene effects and have not considered how combinations of genetic variants and environmental exposures jointly influence osteoporosis risk, particularly in Mexican women, an underrepresented population in genetic osteoporosis research. This approach could also help explain the inconsistencies in results reported across population-based studies on osteoporosis risk.

Given the inconsistent associations of *CYP19A1*, *ESR1*, *IL6*, *IL6R*, *IL1β*, *RANK* and *RANKL* genes across populations and the limited research examining interactions effects, we aimed to evaluate both individual SNVs and gene–gene and gene–environment interactions in relation to OP and HFx risk in Mexican women.

## 2 Methods

### 2.1 Subjects

A total of 609 postmenopausal Mexican mestizo women were recruited from the Osteoporosis Clinic and Traumatology Department at the Instituto Nacional de Rehabilitación Luis Guillermo Ibarra Ibarra. The study population included 169 women with OP, 235 women with osteoporotic fragility fracture of the hip (HFx), and 205 controls (defined as women without OP, HFx, or osteopenia), all aged 45 years or older. All participants provided informed consent to participate voluntarily in the study. All participants were women of Mexican mestizo origin, defined as individuals born in Mexico with a Spanish-derived surname and at least three generations of documented mestizo ancestry. None of the participants were biologically related, and all were born in the central, southern, or southeastern regions of Mexico, consistent with the ancestry of their maternal and paternal lineages. Women who did not meet the Mexican mestizo ancestry criteria, and those with fractures unrelated to OP, were excluded from the study.

The initial calculation of the sample size was performed for an unmatched case-control study. This calculation was based on the prevalence of 18% of the minor allele frequency (MAF) of the rs1800795 single nucleotide polymorphism, as reported for the Mexican population in the 1000 Genomes Project Phase 3 allele frequencies. Using these data and setting the statistical parameters at an alpha level of 0.05, a beta of 0.80, and a two-tailed test, it was determined that a minimum of 182 participants per group would be required to detect statistically significant differences. The rs1800795 variant was selected for this analysis because it has previously been associated with osteoporosis in Mexican women, as documented by Villagómez Vega et al. (2023).

Classification into OP and control groups were performed according to World Health Organization (WHO) criteria, based on densitometric analysis of the femoral neck and lumbar spine using a Hologic 2000 device (Hologic, Inc., San Francisco, CA, United States). Hip fractures were confirmed radiographically.

### 2.2 DNA and genotyping

Genomic DNA was extracted from 5 mL of peripheral blood using the PUREGENE DNA Extraction Kit and PUREGENE Blood Core Kit (Qiagen, Hilden, Germany), following the manufacturer’s instructions. Genotyping was performed using TaqMan probes (Applied Biosystems, Foster City, CA, United States). Reactions were run on a StepOne Real-Time PCR System (Applied Biosystems) in a final volume of 25 µL containing 1x TaqMan PCR Master Mix, 100 nM of the specific probe, 900 nM of each primer, and 25 ng of genomic DNA. Cycling conditions included an initial denaturation step at 95 °C for 10 min, followed by 40 cycles of denaturation at 92 °C for 15 s and annealing/extension at 60 °C for 1 min. A total of 14 SNVs were genotyped. These were selected from OP and fracture association studies previously published (https://pubmed.ncbi.nlm.nih.gov/). Their characteristics are provided in Table 1.

**TABLE 1:** Characteristics of the single nucleotide variants analyzed.

Gene and SNV	Position	Alleles	HHF4jb
*CYP19*			
rs700518	chr15:51236915	T>C	Synonymous variant
rs1062033	chr15:51255741	C>G	Intron variant
rs4775936	chr15:51243825	C>T	Intron variant
rs767199	chr15:51248190	G>A	Intron variant
rs17523880	chr15:51300346	C>A	Intron variant
*ESR1*			
rs2234693	chr6:151842200	T>C	Intron variant
rs2228480	chr6:152098960	G>A	Synonymous variant
*IL6*			
rs1800795	chr7:22727026	C>G	Intron variant
rs1800796	chr7:22726627	G>C	Intron variant
*IL6R*			
rs4845617	chr1:154405422	G>A	5′UTR variant
rs2228145	chr1:154454494	A>C	Missense variant
*IL1B*			
rs16944	chr2:112837290	A>G	Upstream transcript variant
*RANK*			
rs3018362	chr18:62414860	A>G	None
*RANK-L*			
rs12585014	chr13:42566423	G>A	Intron variant

### 2.3 Statistical analysis

Descriptive statistics were performed and continuous variables were compared using Student’s t-test or the Mann–Whitney U-test, depending on data distribution. For categorical variables, Chi-squared (χ^2^) or Fisher’s exact tests were applied. For each gene variant, the allelic and genotypic frequencies were calculated, and Hardy–Weinberg equilibrium (HWE) was assessed.

All single nucleotide variants (SNVs) included in the study were successfully genotyped across all participants. As a result, there was no missing genotype data for any of the individuals in the cohort. For the demographic variables, any missing data were addressed using an imputation method. This approach ensured that the dataset remained complete and suitable for subsequent analyses.

Genotypic association analyses were conducted using codominant, dominant, and recessive inheritance models. Univariate and multivariate analyses were performed using non-conditional logistic regression to estimate the risk of developing OP or HFx, considering each genotype as the main effect. Unadjusted and adjusted odds ratios (ORs) with 95% confidence intervals (CIs) were reported. All statistical analyses were performed using STATA version 15.0 (software package, Stata Corporation, College Station, TX, United States).

Additionally, the 10-year fracture risk was estimated using the FRAX® algorithm (https://www.fraxplus.org/), which calculates the probability of hip and major osteoporotic fractures based on clinical risk factors with and without BMD for subjects in the OP and control groups and without BMD for the HFx group. These scores were included as environmental variables in the gene–environment interaction analyses.

Haplotypes of SNVs in the *CYP19A1, ESR1* and *IL6R* genes were constructed, and their associations with OP and HFx were analyzed using Haploview version 4.1.

Identifying gene variations associated with complex diseases is a challenge because this is likely to be the result of many genetic and environmental factors interactions that can play a crucial role in the development of the diseases. Multifactor dimensionality reduction (MDR) is useful to address these concerns. This is a non-parametric and model free method designed to detect gene–gene or gene–environment interactions that could confer disease risk. MDR 3.0.2 software and the full MDR procedure is available at epistasis.org. Briefly, the dataset is randomly divided into 10 subsets. From the pool of genetic and environmental factors, a set of *n* factors is selected, and all possible combinations are evaluated for their ability to classify cases and controls using 9/10 of the data (training set) to select the best *n*-factor model (including genes and/or environment factors). The remaining 1/10 of the data (testing set) is used for independent testing for cross validation consistency (CVC). This process is repeated 10 times with the data split into 10 different training and testing sets. The best models are identified based on their testing accuracy (proportion of correctly classified cases and controls) and CVC, defined as the number of times a particular set of factors is identified in each possible 9/10 of the subjects. The best model is that with the highest CVC and testing accuracy values since this model shows more consistent results. ORs with 95% CIs are calculated (Moore et al., 2006; Motsinger and Ritchie, 2006).

## 3 Results

A total of 609 women were included in this study: 169 with OP, 205 with HFx, and 235 controls. Characteristics of the study population are summarized in Table 2. The mean age was 57.78 ± 8.44 years in the control group, 69.62 ± 9.88 years in the OP group, and 77.7 ± 10.89 years in the HFx group. The mean body mass index (BMI, kg/m^2^) was 28.76 ± 4.01 in the control group, 25.05 ± 3.99 in the OP group, and 24.87 ± 4.6 in the HFx group. Variables such as age at menarche, number of pregnancies, and estrogen intake showed significant differences between groups, while age at menopause and smoking habits did not. Regarding comorbidities, hypertension and diabetes differed between the OP and HFx groups compared with controls. The incidence of arthritis also differed significantly between the OP and control groups (Table 2).

**TABLE 2:** Characteristics of cases and controls.

Variable	Controls (n = 205)	Osteoporosis (n = 169)	*p* value	Hip fracture (n = 235)	*p* value
Age (mean ± SD; years)^a^	57.78 ± 8.44	69.62 ± 9.88	**0.0001**	77.7 ± 10.89	**0.0001**
BMI (kg/m^2^)^a^	28.76 ± 4.01	25.05 ± 3.99	**0.0001**	24.87 ± 4.6	**0.0001**
Age at menarche (years)^a^	12.76 ± 1.53	13.34 ± 1.54	**0.0004**	13.08 ± 1.53	**0.034**
Age at menopause (years)^a^	46.96 ± 6.75	45.85 ± 6.52	0.117	46.66 ± 5.82	0.627
Pregnancies^b^	3 (0–12)	3 (0–14)	0.085	5 (0–17)	**0.0001**
Estrogens intake^c^	40 (19.61)	35 (20.71)	0.791	16 (6.9)	**0.0001**
Smoking habit^c^	45 (21.95)	26 (15.38)	0.107	39 (16.74)	0.167
Hypertension^c^	50 (24.39)	70 (41.67)	**0.0001**	115 (49.57)	**0.0001**
Diabetes^c^	19 (9.27)	28 (16.67)	0.032	68 (29.06)	**0.0001**
Arthritis^c^	14 (6.83)	25 (14.88)	0.011	13 (5.60)	0.595
FRAX major without BMD^b^^,^^&^	3.2 (2.3–6.1)	11 (6.3–17)	**0.0001**	16.0 (11.0–22.0)	**0.0001**
FRAX hip without BMD^b^^,^^&^	0.4 (0.2–1.2)	4.1 (1.5–8.6)	**0.0001**	7.6 (4.4–12)	**0.0001**
FRAX major^#^ with BMD^b^^,^^&^	2.7 (2.0–4.5)	13 (9.4–17)	**0.0001**	—	—
FRAX hip^#^ with BMD^b^^,^^&^	0.1 (0.1–0.4)	5.2 (3.5–7.9)	**0.0001**	—	—

^a^Expressed as mean ± standard deviation.

^b^Expressed as median (minimum-maximum values).

^c^Expressed as number of individuals (%).

^#^The FRAX® algorithms give the 10-year probability of hip fracture and the 10-year probability of a major osteoporotic fracture (clinical spine, forearm, hip or shoulder fracture), with and without BMD.

^&^Bone mineral density. Significant values are shown in bold.

Regarding fracture risk predictions using the FRAX tool, the 10-year probability of a major fracture without BMD was significantly higher in both the OP and HFx groups compared with controls (p = 0.0001 in both cases). Although the 10-year probability of a major fracture based on BMD was not calculated for the HFx group, significant differences were observed between the OP and control groups (p = 0.0001) (Table 2).

All SNVs were in HWE (p > 0.05), except for rs17523880 in *CYP19A1* and rs1800796 in *IL6*. The A allele of *RANK* rs3018362 was significantly more frequent in OP cases than in controls (51.0% vs. 42.0%) and was associated with increased OP risk (OR = 1.5, 95% CI: 1.1–1.9, p = 0.007). No other alleles showed significant differences in distribution between the OP or HFx groups and controls (Table 3).

**TABLE 3:** Allele frequency and association of single nucleotide variants (SNV) in the *CYP19*, *ESR1*, *IL6*, *IL6R*, *IL1B*, *RANK* and *RANKL* genes, with osteoporosis and hip fracture.

SNV alleles	Controls (n = 205) N (%)	OP (n = 169)			HFx (n = 235)			HWE
		N (%)	OR (95% CI)^a^	p^a^	N (%)	OR (95% CI)^b^	p^b^	
*CYP19*								
rs700518								
C	117 (29.0)	92 (27.0)	0.9 (0.7–1.3)	0.68	110 (23.0)	0.8 (0.6–1.03)	0.08	0.14
T	293 (71.0)	246 (73.0)	1.1 (0.8–1.5)	​	360 (77.0)	1.3 (0.9–1.8)	​	​
rs1062033								
G	102 (25.0)	82 (24.0)	0.9 (0.7–1.3)	0.84	99 (21.0)	0.8 (0.6–1.1)	0.17	0.22
C	308 (75.0)	256 (76.0)	1.03 (0.7–1.4)	​	371 (79.0)	1.2 (0.9–1.7)	​	​
rs4775936								
T	109 (27.0)	93 (28.0)	1.05 (0.7–1.4)	0.77	107 (23.0)	0.8 (0.6–1.1)	0.19	0.11
C	363 (77.0)	245 (72.0)	0.9 (0.7–1.3)	​	363 (77.0)	1.2 (0.9–1.7)	​	​
rs767199								
A	110 (27.0)	86 (25.0)	0.9 (0.7–1.3)	0.66	105 (22.0)	0.8 (0.6–1.1)	0.12	0.25
G	300 (73.0)	252 (75.0)	1.1 (0.8–1.5)	​	365 (78.0)	1.3 (0.9–1.7)	​	​
rs17523880								
A	43 (10.0)	42 (12.0)	1.2 (0.7–1.9)	0.40	42 (9.0)	0.8 (0.5–1.3)	0.40	0.0000
C	367 (90.0)	296 (88.0)	0.8 (0.5–1.3)	​	428 (91.0)	1.2 (0.8–1.9)	​	​
*ESR1*								
rs2234693								
C	136 (33.0)	99 (29.0)	0.8 (0.6–1.1)	0.25	130 (28.0)	0.8 (0.6–1.02)	0.07	0.08
T	274 (67.0)	239 (71.0)	1.2 (0.9–1.6)	​	340 (72.0)	1.3 (0.9–1.7)	​	​
rs2228480								
A	118 (29.0)	99 (29.0)	1.02 (0.7–1.4)	0.87	154 (33.0)	1.2 (0.9–1.6)	0.20	0.49
G	292 (71.0)	239 (71.0)	0.9 (0.7–1.3)	​	316 (67.0)	0.8 (0.6–1.1)	​	​
*IL6*								
rs1800795								
C	50 (12.0)	40 (12.0)	0.9 (0.6–1.5)	0.88	45 (10.0)	0.8 (0.5–1.1)	0.21	0.97
G	360 (88.0)	298 (88.0)	1.03 (0.7–1.6)	​	425 (90.0)	1.3 (0.8–2.0)	​	​
rs1800796								
C	147 (36.0)	124 (37.0)	1.03 (0.8–1.4)	0.81	181 (39.0)	1.1 (0.8–1.5)	0.41	0.04
G	263 (64.0)	214 (63.0)	0.9 (0.7–1.3)	​	289 (61.0)	0.9 (0.7–1.2)	​	​
*IL6R*								
rs4845617								
A	201 (49.0)	155 (46.0)	0.9 (0.7–1.2)	0.38	234 (50.0)	1.0 (0.8–1.3)	0.82	0.63
G	209 (51.0)	183 (54.0)	1.1 (0.8–1.5)	​	236 (50.0)	0.9 (0.7–1.3)	​	​
rs2228145								
A	190 (46.0)	146 (43.0)	0.9 (0.6–1.2)	0.38	212 (45.0)	0.9 (0.7–1.2)	0.70	0.09
C	220 (54.0)	192 (57.0)	1.1 (0.8–1.5)	​	258 (55.0)	1.05 (0.8–1.4)	​	​
*IL1B*								
rs16944								
G	170 (41.0)	135 (40.0)	0.9 (0.7–1.2)	0.67	179 (38.0)	0.9 (0.7–1.1)	0.31	0.72
A	240 (59.0)	203 (60.0)	1.1 (0.8–1.4)	​	291 (62.0)	1.1 (0.9–1.5)	​	​
*RANK*								
rs3018362								
A	171 (42.0)	174 (51.0)	**1.5 (1.1–1.9)**	**0.007**	219 (47.0)	1.2 (0.9–1.6)	0.15	0.44
G	239 (58.0)	164 (49.0)	**0.7 (0.5–0.9)**	​	251 (53.0)	0.8 (0.6–1.1)	​	​
*RANKL*								
rs12585014								
A	161 (39.0)	133 (39.0)	1.0 (0.7–1.3)	0.9	186 (40.0)	1.01 (0.8–1.3)	0.90	0.11
G	249 (61.0)	205 (61.0)	0.9 (0.7–1.3	​	284 (60.0)	0.9 (0.7–1.3)	​	​

OP, osteoporosis; HFx, hip fracture; CI, confidence intervals; OR, odds ratio; ^a^OP, vs. controls; ^b^HFx, vs. controls; HWE, Hardy-Weinberg equilibrium; significant values are shown in bold.

Genotypic analysis revealed that *RANK* rs3018362 was the most significantly associated SNV with OP. Under the codominant model, both GA and AA genotypes were associated with increased risk in unadjusted analyses, and these associations remained significant after adjustment (Adjusted OR = 2.08, 95% CI: 1.08–3.98 for GA; and 2.76, 95% CI: 1.21–6.30 for AA). The dominant model also yielded a significant association (Adjusted OR = 2.23, 95% CI: 1.19–4.17). For *ESR1* rs2234693, the CC genotype was associated with a reduced OP risk under both codominant and recessive models, but only in the adjusted analysis (Adjusted OR = 0.28, 95% CI: 0.11–0.69 and 0.25, 95% CI: 0.10–0.61, respectively) (Supplementary Table S1A).

In the HFx group, the CC genotype of *CYP19A1* rs700518 was associated with lower fracture risk in both the codominant and recessive models (OR = 0.45, 95% CI: 0.21–0.96 and OR = 0.47, 95% CI: 0.22–0.98, respectively), although these associations lost significance after adjustment. The CC genotype of *ESR1* rs2234693 remained significantly associated with reduced fracture risk in the adjusted recessive model (OR = 0.30, 95% CI: 0.12–0.75). Conversely, the AA genotype of *RANK* rs3018362 was associated with increased fracture risk in the adjusted codominant model (OR = 2.4, 95% CI: 1.01–6.06) (Supplementary Table S1B).

Haplotypes of *CYP19A1*, *ESR1*, and *IL6R* were constructed separately for each gene to assess their associations with OP and HFx. However, no statistically significant associations were observed (Table 4).

**TABLE 4:** Haplotypes of CYP19, ESR1 and IL6R variants in osteoporosis and hip fracture.

Haplotype	Case, control frequencies	OR (95% CI)	P value
Osteoporosis			
*CYP19*			
rs700518/rs4775936/rs767199/rs1062033			
TCGC	0.713, 0.692	1.1 (0.8–1.5)	0.54
CTAG	0.239, 0.227	1.1 (0.7–1.5)	0.68
CTGC	0.018, 0.012	1.5 (0.4–4.8)	0.53
TTGC	0.012, 0.010	1.2 (0.3–4.8)	0.78
CCAC	0.003, 0.017	0.2 (0.02–1.4)	0.06
*ESR1*			
rs2234693/rs2228480			
TG	0.475, 0.447	1.1 (0.8–1.5)	0.44
CG	0.232, 0.265	0.8 (0.6–1.2)	0.30
TA	0.232, 0.221	1.1 (0.7–1.5)	0.73
CA	0.061, 0.066	0.9 (0.5–1.6)	0.75
*IL6R*			
rs4845617/rs2228145			
AC	0.358, 0.372	0.9 (0.7–1.3)	0.70
GA	0.332, 0.345	0.9 (0.7–1.3)	0.70
GC	0.210, 0.165	1.3 (0.9–1.9)	0.12
AA	0.100, 0.119	0.8 (0.5–1.3)	0.43
Hip fracture			
*CYP19*			
rs700518/rs4775936/rs767199/rs1062033			
TCGC	0.751, 0.692	1.3 (0.9–1.8)	0.05
CTAG	0.198, 0.227	0.8 (0.6–1.2)	0.29
CTGC	0.017, 0.012	1.4 (0.5–4.3)	0.57
CCAC	0.011, 0.017	0.6 (0.2–1.9)	0.40
*ESR1*			
rs2234693/rs2228480			
TG	0.451, 0.444	1.0 (0.8–1.3)	0.84
TA	0.272, 0.224	0.5 (0.3–0.6)	0.10
CG	0.221, 0.268	0.8 (0.6–1.1)	0.11
CA	0.055, 0.064	0.9 (0.5–1.5)	0.60
*IL6R*			
rs4845617/rs2228145			
AC	0.401, 0.378	1.1 (0.8–1.4)	0.49
GA	0.354, 0.351	1.1 (0.8–1.3)	0.93
GC	0.148, 0.159	0.9 (0.6–1.3)	0.66
AA	0.097, 0.112	0.8 (0.6–1.3)	0.46

**FIGURE 1:**
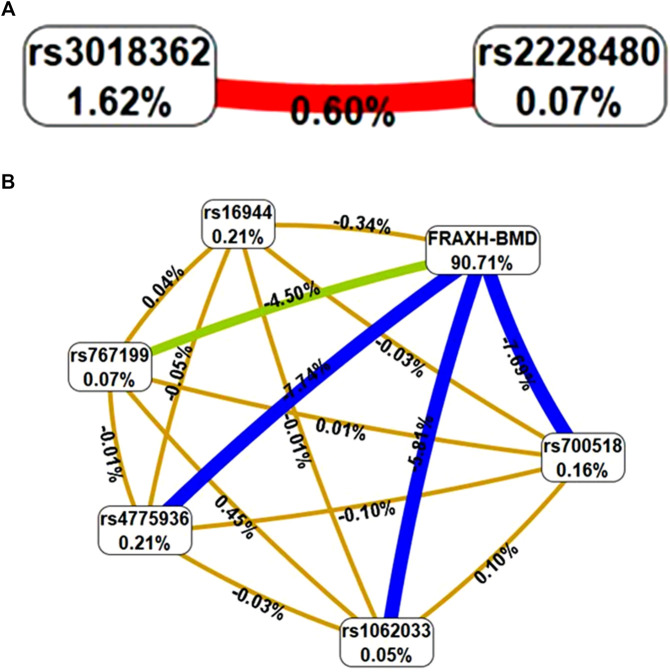
Interaction graph generated through MDR software that describe the percentage of entropy or information gain (IG) in OP case-control status that is explained by each factor. Values on nodes indicate IG of independent effect of each factor, whereas interaction between two factors are depicted by line accompanied by a percent of IG explained by that interaction. Positive IG values between the nodes indicate synergistic interactions. Negative IG values indicate the redundancy between the respective variants. Colors represents a continuum from synergy to redundancy. Red represents the highest degree of interaction or synergy. Orange represents moderate synergy. Golden yellow represent minimal synergy. Green represents very low interaction. Blue means redundancy or no interaction. **(A)** The graph in this two-locus model showed that there was interaction between ESR1 (rs2228480) and RANK (rs3018362); **(B)** Gene-environment interactions between CYP19 (rs700518), CYP19 (rs1062033), CYP19 (rs4775936), CYP19 (rs767199), IL1B (rs16944) and the FRAXH-BMD (10-year probability of hip fracture).

**FIGURE 2:**
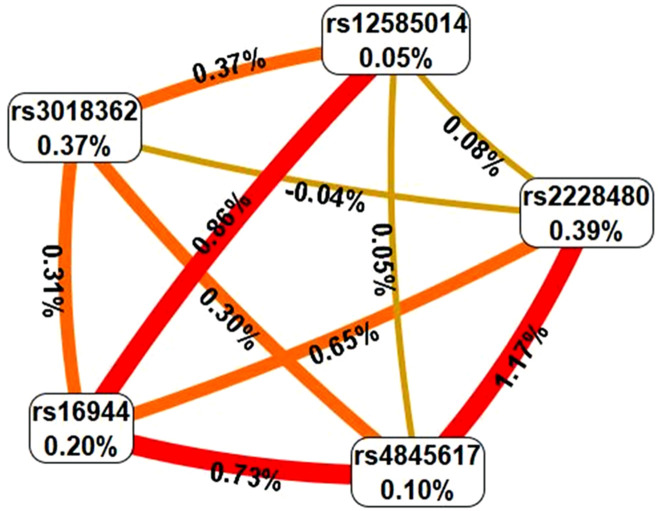
Interaction graph generated through MDR software that describe the percentage of entropy or information gain (IG) in HFx case-control status that is explained by each factor. Explanation of percent values and colors are as in Figure 1. Graph shows gene–gene interaction among *ESR1* (rs2228480)*, IL6R* (rs4845617)*, IL1B* (rs16944), *RANK* (rs3018362), and *RANKL* (rs12585014). No significant gene–environment interactions were observed.

In the MDR analysis, a significant gene–gene interaction was identified between *ESR1* rs2228480 and *RANK* rs3018362 in OP, with a testing accuracy of 0.5656 and a CVC of 10/10. This interaction was also associated with increased OP risk (OR = 2.1, 95% CI: 1.4–3.2) (Table 5; Figure 1, panel A). In the HFx group, the top gene–gene interaction model included *ESR1* (rs2228480), *IL6R* (rs4845617), *IL1β* (rs16944), RANK (rs3018362), and *RANK-L* (rs12585014), with a testing accuracy of 0.5577 and a CVC of 10/10 (Table 6; Figure 2). A significant gene–environment interaction was observed in OP, involving *CYP19A1* SNVs (rs700518, rs1062033, rs4775936, and rs767199), *IL1β* (rs16944), and 10-year probability of hip fracture (FRAXH-BMD). This model yielded a testing accuracy of 0.8488 and a CVC of 10/10 (Table 5; Figure 1, panel B). No significant gene–environment interactions were detected in the HFx group (Table 6).

**TABLE 5:** Multifactor dimensionality reduction interaction model in the osteoporosis.

Model	Training accuracy	Testing accuracy	CVC	OR (95% CI)	P
Gene-gene interaction					
*ESR1* (rs2228480), *RANK* (rs3018362)	0.5894	0.5656	10/10	2.1 (1.4–3.2)	0.0006
*IL6R* (rs4845617), *IL6R* (rs2228145), *IL1B* (rs16944)	0.6297	0.4662	2/10	2.8 (1.8–4.3)	0.0001
*IL6R* (rs4845617), *IL1B* (rs16944), *RANK* (rs3018362), *RANKL* (rs12585014)	0.6951	0.437	3/10	4.7 (2.9–7.3)	0.0001
Gene-environment interaction					
BMI, FRAX- noBMD	1.0	0.5507	8/10	NA	0.0001
*CYP19* (rs700518), Age, FRAX-BMD	1.0	0.5727	8/10	NA	0.0001
*CYP19* (rs700518), *CYP19* (rs1062033), Age, FRAX-HBMD	1.0	0.5854	10/10	NA	0.0001
*CYP19* (rs700518), *CYP19* (rs1062033), *CYP19* (rs4775936), Age, FRAX-HBMD	1.0	0.5829	10/10	NA	0.0001
*CYP19* (rs700518), *CYP19* (rs1062033), *CYP19* (rs4775936), *CYP19* (rs767199), *IL1B* (rs16944), FRAX-HBMD	1.0	0.8488	10/10	NA	0.0001

CVC, cross-validation consistency, which shows how often the best model was found across the different cross-validation subsets; a CVC, of 10/10 indicates more consistent results. 10-year probability of major fracture. The best model is that with the highest testing accuracy and CVC, values. NA, not available; the risk could not be could not calculated because of the existence of empty data cells.

**TABLE 6:** Multifactor dimensionality reduction interaction model in hip fracture.

Model	Training accuracy	Testing accuracy	CVC	OR (95% CI)	P
Gene-gene interaction					
*ESR1* (rs2228480), *IL6R* (rs4845617)	0.5716	0.4615	3/10	2.1 (1.3–3.3)	0.0015
*ESR1* (rs2228480), *IL6R* (rs4845617), *IL1B* (rs16944)	0.6183	0.4837	2/10	2.5 (1.7–3.6)	0.0001
*ESR1* (rs2228480), *IL6R* (rs4845617), *IL1B* (rs16944), *RANKL* (rs12585014)	0.6907	0.5635	8/10	4.7 (3.2–7.1)	0.0001
*ESR1* (rs2228480), *IL6R* (rs4845617), *IL1B* (rs16944), *RANK* (rs3018362), *RANKL* (rs12585014)	0.7725	0.5577	10/10	10.3 (6.5–16.5)	0.0001
Gene-environment interaction					
Age, BMI	0.9965	0.5372	9/10	NA	0.0001
*ESR1* (rs2228480), Age, BMI	1.0	0.5012	2/10	NA	0.0001
*CYP19* (rs700518), *ESR1* (rs2228480), Age, BMI	1.0	0.5037	2/10	NA	0.0001

CVC, cross-validation consistency; which shows how often the best model was found across the different cross-validation subsets; a CVC, of 10/10 indicates more consistent results. The best model is that with the highest testing accuracy and CVC, values. NA, not available; the risk could not be could not calculated because of the existence of empty data cells.

## 4 Discussion

Our results demonstrate an association between variants in *ESR1* and *RANK* and the risk of both OP and HFx. The *ESR1* SNVs have been implicated in numerous conditions including cartilage and bone diseases. Variants rs9340799 and rs2228480 are associated with risk of osteoarthritis, but rs2234693 was not in a meta-analysis from many populations throughout the world (Ma et al., 2015). In Mexican population rs2234693 and rs9340799 SNVs, were associated with a reduced risk of osteoarthritis (Borgonio-Cuadra et al., 2012).

With regard to OP and Fx, the variant rs2234693 has previously been associated with higher T-scores in postmenopausal Slovak women (Mondockova et al., 2018), and with increased femoral neck BMD and Z-scores in Asian populations (Zhu et al., 2018).

Particularly in Mexican population this variant has been linked to a reduced risk of OP and HFx in Mexican women, (García-Rojas et al., 2022), and of distal radius fracture (Farías et al., 2020). The variants rs3020331, rs3020404 and rs4870044 showed association with a reduced risk of osteoporosis and/or hip fracture in a cohort of Mexican women (Hidalgo-Bravo et al., 2019). These results are consistent with our actual findings showing the protective association of *ESR1* SNVs in independent-effect analyses. There is one study with Mexican postmenopausal women, were the rs2234693 did not show association with BMD variations (Rojano-Mejía et al., 2014), However, in Mexican population with only limited exceptions *ESR1* variants are usually associated as protective factors against low BMD and osteoporosis.

Variants of the *RANK/RANKL/OPG* genes have showed association with several bone conditions. For example, *RANK* rs3826620 and *RANKL* rs9594738 were associated with periodontitis (Petean et al., 2019). Genetic variants of *RANK*/*RANKL/OPG* increase the risk of rheumatoid arthritis in Mexican individuals (Nava-Valdivia et al., 2024) and Chinese populations (Pei et al., 2025) and the *OPG* rs2073618 is related with increased risk of Legg-Calvé-Perthes disease in Mexican children (Cruz-Ortíz et al., 2024).

With regard to osteoporosis and fragility fractures, previous reports associated the *RANK* rs3018362 SNV with reduced cortical BMD, increased bone resorption, with low-trauma and atraumatic fractures in several Caucasian populations (Chen et al., 2003; Kemp et al., 2014; Styrkarsdottir et al., 2008). In Mexican women, it was reported association between this variant and elevated OP risk (Casas-Avila et al., 2019). In the present study, was associated with increased risk of OP and HFx in Mexican women, consistent with previous reports that support the role of *RANK* rs3018362 as a risk variant across diverse populations.

The variants in *IL6* gene explored in this work were not associated with OP or Fx in the individual analysis. As occurred in previous investigations with Mexican population, where the rs1800795 variant of *IL6* did not showed association with risk of fracture (Ponce de León-Suárez et al., 2018) nor with BMD variations in postmenopausal obese women (Méndez et al., 2013). However, it showed an important association with severe radiographic damage of the hands in patients with rheumatoid arthritis in Mexican adult patients (Zavaleta-Muñiz et al., 2016). In the other hand, the rs1800796 (GG genotype) of *IL6* has been associated with reduced risk of hip fracture in Mexican women (Ponce de León-Suárez et al., 2018) but it was not associated in this study. Few studies examined the effects of IL6R variants on OP in Mexican population, but the SNVs rs4845617 and rs2228145 were not associated with hip fragility fracture in postmenopausal Mexican women (Ponce de León-Suárez et al., 2018) just as happened in this study.

Aromatase contributes importantly to bone metabolism. Variants in *CYP19A1* gene have been studied with regard to the risk of vertebral fractures (Koudu et al., 2012) and with regard to the BMD in women with hormonal replacement therapy (Masi et al., 2014). In Mexican population the rs700518 was associated to response to pharmacological treatment for OP (Villagómez Vega et al., 2023) and (TTTA)n microsatellite combined with a TCT deletion were associated to HFx risk (Casas-Avila et al., 2015), but they have not been widely studied in Mexican. As well as IL6 and IL6R variants, *CYP19A1* SNVs were not associated directly with OP or HFx in this study; however, they associate when interact with other factors, emphasizing the value of interaction analyses for detecting risk-modifying effects that are not apparent in single-variant models.

To the best of our knowledge, no prior study has simultaneously evaluated all these genes and comprehensively examined both gene–gene and gene–environment interactions in the context of OP and HFx risk. Epistasis, defined as gene–gene interactions between two or more loci, may alter disease risk independently of individual gene effects (Perelygin et al., 2025). Recent research has explored the role of epistasis in OP. For example, an interaction between *RMBS3* and *ZNF516* has been shown to influence BMD in Caucasian and African populations (Yang et al., 2013), while rs3751143 of *P2X7R* interacting with *ESR1* rs2234693 increases OP susceptibility in postmenopausal Chinese women (Wang et al., 2018). Additionally, a *VDR*–*TNF-α* interaction has been associated with OP risk in elderly women (Liao et al., 2019). In our study, a significant gene–gene interaction between *ESR1* and *RANK* was observed in women with OP, as well as a gene–environment interaction involving *CYP19A1*, *IL1β*, and the 10-year probability of major fractures. Although in the graphic representation might seem that there is no gene-environment interaction in OP (Figure 1B), positive IG values between nodes and values in Table 5 for the best model, show synergistic interactions between these factors. For HFx, a gene–gene interaction was identified involving *ESR1, IL6R, IL1β, RANK and RANKL,* while no significant gene–environment interactions were detected in this group.

Bone cells, including osteocytes, osteoclasts, and osteoblasts, are major targets of estrogen. Estrogens, through their receptors (ESR), contribute to bone homeostasis by reducing bone resorption, partly through inhibition of RANKL expression in osteoblasts and increased production of osteoprotegerin (OPG). However, this protective mechanism declines after menopause, when bone resorption begins to exceed bone formation (Fischer and Haffner-Luntzer, 2022). The interaction observed between *ESR1* and *RANK* in our study may indicate that in postmenopausal women, the role of *ESR1* shifts from protective to a risk-modifying factor when interacts with *RANK*. Notably, this interaction involved SNV’s that were not significant when analyzed independently.

In HFx, the interaction among *ESR1, IL6R*, *IL1β*, *RANK* and *RANKL* genes conferred a substantially increased fracture risk. It can be explained because *IL1β* and *IL6,* and their respective receptors, are key mediators of inflammatory signaling and bone resorption in osteoporosis and other diseases leading bone damage (Tseng et al., 2022; Takeuchi et al., 2021). As estrogen deficiency also downregulates OPG and increases *RANKL* activity, the combined effect of proinflammatory and osteoclast-activating signals may account for the high fracture susceptibility observed in the presence of these interacting variants.

We also identified a gene–environment interaction in OP involving *CYP19A1*, and the 10-year probability of major fractures, in addition to *IL1β* (Table 6). This finding is biologically plausible, as aromatase encoded by *CYP19A1* gene, is responsible for converting androgens to estrogens, a crucial source of estrogens in postmenopausal women, and the rs700518 and rs1062033 *CYP19A1* variants have been previously associated with reduced expression (Riancho et al., 2007). All these facts likely may contribute to OP in our sample.

We recognized that our study could have some limitations. As a hospital-based case–control study, it carries an inherent risk of selection bias. To address this concern, we took comprehensive steps to minimize bias in our research design. To avoid selection bias, we carefully assessed cases with OP and HFx alongside their corresponding controls. This was accomplished by applying specific inclusion criteria for both groups, ensuring that comparisons were valid and reliable. Besides, we also control potential confounders. This was achieved through the use of exclusion criteria and by applying multivariate statistical analysis during data evaluation. These measures enhanced the accuracy of our findings by reducing the influence of variables outside the scope of our study. Population stratification is a known complication in association studies involving admixed populations. Given that Mexicans represent an admixed population, we took steps to limit stratification effects. Specifically, cases and controls were selected from particular regions of Mexico to minimize the impact of subpopulation differences. Additionally, we utilized a validated questionnaire to assess ethnic ancestry and anthropometric variations among participants. In summary, while we acknowledge the limitations inherent in our study, we are confident that the main strength of our work lies in the careful and appropriate selection of study groups. This approach enhances the overall validity and reliability of our research findings.

## 5 Conclusion

Our findings indicate that both individual effect of genetic variants as well as gene–gene and gene–environment interactions contribute to OP and HFx susceptibility in Mexican women. These results highlight the importance of evaluating polygenic and environmental interactions rather than isolated genetic effects, particularly in populations with distinct complex ancestral backgrounds.

This study contributes to a more comprehensive understanding of osteoporosis genetics in Mexican women. Future studies with larger and multi-ethnic cohorts are necessary to validate and expand upon these findings.

## Data Availability

The datasets presented in this study can be found in online repositories with the following accession link: https://doi.org/10.5281/zenodo.18761470.
