# Supplementary material for: Gene-gene and gene-environment interactions of CYP19A1, ESR1, IL6, IL6R, IL1β, RANK, and RANKL variants in relation to osteoporosis and hip fracture risk in Mexican women
Source: Front Aging. 2026 Mar 3;7:1769306. doi: 10.3389/fragi.2026.1769306 (PMC13040349; doi:10.3389/fragi.2026.1769306)
Supplement: Supplementary file 2 [file Table1.docx]

SUPPLEMENTARY TABLE 1 (A and B).

Supplementary Table 1 A. Genotypic frequencies and association of SNV in the *CYP19*, *ESR1*, *IL6*, *IL6R*, *IL1B*, *RANK* and *RANKL* genes, with osteoporosis.

| **SNP and**  **Model** | **Controls**  **n (%)** | **OP**^a^  **n (%)** | **OR (95% CI)^b^** | **p-value**^b^ | **OR (95% CI)^c^** | **p-value**^c^ |
| --- | --- | --- | --- | --- | --- | --- |
| ***CYP19***  **rs700518**  Codominant  TT  TC  CC | 109 (53.7)  75 (36.59)  21 (10.24) | 90 (53.25)  66 (39.05)  13 (7.69) | 1.00  1.106 (0.69-1.64)  0.74 (0.35-1.58) | 0.773  0.449 | 0.767 (0.43-1.36)  0.706 (0.27-1.79) | 0.364  0.464 |
| Dominant  TC/CC vs TT | 96 (46.83) | 79 (46.75) | 0.99 (0.66-1.49) | 0.987 | 0.75 (0.44-1.28) | 0.302 |
| Recessive  CC vs TC/TT | 21 (10.24) | 13 (7.69) | 0.73 (0.35-1.50) | 0.394 | 0.78 (0.32-1.93) | 0.602 |
| **rs1062033**  Codominant  CC  CG  GG | 119 (58.05)  70 (34.15)  16 (7.80) | 98 (57.99)  60 (35.50)  11 (6.51) | 1.00  1.04 (0.67-1.61)  0.83 (0.37-1.88) | 0.857  0.663 | 0.59 (0.33-1.07)  0.85 (0.30-2.39) | 0.088  0.762 |
| Dominant  CG/GG vs CC | 86 (41.95) | 71 (42.01) | 1.00 (0.66-1.51) | 0.991 | 0.63 (0.36-1.11) | 0.113 |
| Recessive  GG vs CG/CC | 16 (7.80) | 11 (6.51) | 0.82 (0.37-1.82) | 0.630 | 1.02 (0.37-2.77) | 0.966 |
| **rs4775936**  Codominant  CC  CT  TT | 115 (56.10)  71 (34.63)  19 (9.27) | 89 (52.66)  67 (39.64)  13 (7.69) | 1.00  1.21 (0.79-1.88)  0.81 (0.41-1.88) | 0.370  0.750 | 0.89 (0.50-1.58)  0.79 (0.31-2.01) | 0.700  0.627 |
| Dominant  CT/TT vs CC | 90 (43.90) | 80 (47.34) | 1.14 (0.76-1.72) | 0.507 | 0.87 (0.51-1.48) | 0.613 |
| Recessive  TT vs CC/CT | 19 (9.27) | 13 (7.69) | 0.81 (0.39-1.70) | 0.588 | 0.82 (0.33-2.05) | 0.685 |
| **rs767199**  Codominant  GG  GA  AA | 113 (55.12)  74 (36.10)  18 (8.78) | 95 (56.21)  62 (36.69)  12 (7.10) | 1.00  0.99 (0.64-1.53)  0.79 (0.36-1.72) | 0.988  0.560 | 0.64 (0.36-1.61)  0.66 (0.24-1.77 | 0.145  0.416 |
| Dominant  GA/AA vs GG | 92 (44.88) | 74 (43.79) | 0.95 (0.63-1.44) | 0.833 | 0.65 (0.37-1.12) | 0.125 |
| Recessive  AA vs GG/GA | 18 (8.78) | 12 (7.10) | 0.74 (0.37-1.69) | 0.552 | 0.79 (0.30-2.03) | 0.626 |
| **rs17523880**  Codominant  CC  CA  AA | 179 (87.32)  9 (4.39)  17 (8.29) | 148 (87.57)  0 (0)  21 (12.43) | 1.00  --  1.49 (0.76-2.93) | --  0.244 | --  1.25 (0.54 – 2.94) | --  0.605 |
| Dominant  CA/AA vs CC | 26 (12.68) | 21 (12.43) | 0.97 (0.52-1.80) | 0.941 | 0.94 (0.42-2.06) | 0.880 |
| Recessive  AA vs CC/CA | 17 (8.29) | 21 (12.43) | 1.56 (0.79-3.08) | 0.191 | 1.25 (0.53-2.94) | 0.596 |
| ***ESR1***  **rs2234693**  Codominant  TT  TC  CC | 97 (47.32)  80 (39.02)  28 (13.66) | 85 (50.30)  69 (40.83)  15 (8.88) | 1.00  0.98 (0.63-1.51)  0.61 (0.30-1.22) | 0.943  0.163 | 1.20 (0.68-2.14)  **0.28 (0.11-0.69)** | 0.523  **0.006** |
| Dominant  TC/CC vs TT | 108 (52.68) | 84 (49.70) | 0.88 (0.59-1.33) | 0.566 | 0.87 (0.51-1.48) | 0.616 |
| Recessive  CC vs TT/TC | 28 (13.66) | 15 (8.88) | 0.61 (0.31-1.19) | 0.152 | **0.25 (0.10-0.61)** | **0.002** |
| **rs2228480**  Codominant  GG  GA  AA | 102 (49.76)  88 (42.93)  15 (7.32) | 85 (50.30)  69 (40.83)  15 (8.88) | 1.00  0.94 (0.61-1.44)  1.2 (0.55-2.59) | 0.780  0.643 | 1.04 (0.60-1.82)  1.22 (0.43-3.39) | 0.876  0.703 |
| Dominant  GA/AA vs GG | 103 (50.24) | 84 (49.70) | 0.97 (0.65-1.47) | 0.917 | 1.07 (0.63-1.82) | 0.802 |
| Recessive  AA vs GG/GA | 15 (7.32) | 15 (8.88) | 1.2 (0.58-2.6) | 0.581 | 1.19 (0.44-3.21) | 0.723 |
| ***IL6***  **rs1800795**  Codominant  GG  GC  CC | 158 (77.07)  44 (21.46)  3 (1.46) | 133 (78.70)  32 (18.93)  4 (2.37) | 1.00  0.86 (0.51-1.43)  1.58 (0.34-7.20) | 0.575  0.552 | 0.80 (0.42-1.52)  1.11 (0.18-6.76) | 0.509  0.905 |
| Dominant  GC/CC vs GG | 47 (22.93) | 36 (21.30) | 0.90 (0.55-1.48) | 0.707 | 0.83 (0.44-1.53) | 0.554 |
| Recessive  CC vs GG/GC | 3 (1.46) | 4 (0.85) | 1.63 (0.36-7.38) | 0.525 | 1.17 (0.19-7.06) | 0.860 |
| **rs1800796**  Codominant  GG  GC  CC | 91 (44.39)  81 (39.51)  33 (16.10) | 75 (44.38)  64 (37.87)  30 (17.75) | 1.00  0.95 (0.61-1.50)  1.10 (0.61-1.97) | 0.854  0.741 | 1.09 (0.61-1.95)  1.68 (0.78-3.61) | 0.759  0.183 |
| Dominant  GC/CC vs GG | 114 (55.61) | 94 (55.62) | 1.00 (0.66-1.50) | 0.998 | 1.24 (0.73-2.11) | 0.423 |
| Recessive  CC vs GG/GC | 33 (16.10) | 30 (17.75) | 1.12 (0.65-1.93) | 0.671 | 1.61 (0.78-3.29) | 0.191 |
| ***IL6R***  **rs4845617**  Codominant  GG  GA  AA | 55 (26.83)  99 (48.29)  51 (24.88) | 50 (29.59)  83 (49.11)  36 (21.30) | 1.00  0.92 (0.56-1.49)  0.77 (0.43-1.37) | 0.742  0.387 | 0.83 (0.44-1.54)  0.73 (0.34-1.54) | 0.554  0.411 |
| Dominant  GA/AA vs GG | 150 (73.17) | 119 (70.41) | 0.87 (0.55-1.37) | 0.555 | 0.79 (0.44-1.43) | 0.448 |
| Recessive  AA vs GG/GA | 51 (24.88) | 36 (21.30) | 0.81 (0.50-1.32) | 0.416 | 0.82 (0.44-1.54) | 0.551 |
| **rs2228145**  Codominant  CC  CA  AA | 53 (25.85)  114 (55.61)  38 (18.54) | 50 (30.74)  92 (48.51)  27 (20.85) | 1.00  0.85 (0.53-1.37)  0.75 (0.40-1.40) | -  0.519  0.375 | 1.15 (0.62-2.15)  0.91 (0.39-2.07) | 0.648  0.818 |
| Dominant  CA/AA vs CC | 152 (74.15) | 119 (70.41) | 0.82 (0.52-1.30) | 0.422 | 1.09 (0.60-1.99) | 0.769 |
| Recessive  AA vs CC/CA | 38 (18.54) | 27 (15.98) | 0.83 (0.48-1.43) | 0.516 | 0.82 (0.40-1.66) | 0.587 |
| ***IL1B***  **rs16944**  Codominant  AA  AG  GG | 69 (33.66)  102 (49.76)  34 (16.59) | 64 (37.87)  75 (44.38)  30 (17.75) | 1.00  0.79 (0.50-1.24)  0.95 (0.52-1.72) | 0.314  0.870 | 0.72 (0.39-1.30)  1.32 (0.61-2.84) | 0.282  0.478 |
| Dominant  AG/GG vs AA | 136 (66.34) | 105 (62.13) | 0.83 (0.54-1.27) | 0.397 | 0.85 (0.48-1.48) | 0.571 |
| Recessive  GG vs AA/AG | 34 (16.59) | 30 (17.75) | 1.08 (0.63-1.86) | 0.766 | 1.60 (0.81-3.17) | 0.175 |
| ***RANK***  **rs3018362**  Codominant  GG  GA  AA | 67 (32.68)  105 (51.22)  33 (16.10) | 34 (27.66)  96 (51.49)  39 (20.85) | 1.00  **1.80 (1.09-2.96)**  **2.32 (1.25-4.33)** | **0.020**  **0.008** | **2.08 (1.08-3.98)**  **2.76 (1.21-6.30)** | **0.027**  **0.015** |
| Dominant  GA/AA vs GG | 138 (67.32) | 135 (79.88) | **1.92 (1.19-3.10)** | **0.007** | **2.23 (1.19-4.17)** | **0.012** |
| Recessive  AA vs GG/GA | 33 (16.10) | 39 (20.85) | 1.56 (0.93-2.6) | 0.090 | 1.65 (0.84-3.26) | 0.142 |
| ***RANKL***  **rs12585014**  Codominant  GG  GA  AA | 81 (39.51)  87 (42.44)  37 (18.05) | 64 (37.87)  77 (45.56)  28 (16.57) | 1.00  1.12 (0.71-1.75)  0.95 (0.53-1.72) | 0.620  0.886 | 1.36 (0.76-2.43)  1.31 (0.60-2.86) | 0.299  0.489 |
| Dominant  GA/AA vs GG | 124 (60.49) | 105 (62.13) | 1.07 (0.70-1.62) | 0.746 | 1.34 (0.78-2.31) | 0.278 |
| Recessive  AA vs GG/GA | 37 (18.05) | 28 (16.57) | 0.90 (0.52-1.54) | 0.707 | 1.12 (0.54-2.31) | 0.746 |

a: Osteoporosis; b: unadjusted data; c: adjusted by age, BMI, hypertension and diabetes; significant values are shown in bold.

Supplementary Table 1 B. Genotypic frequencies and association of SNV in the *CYP19*, *ESR1*, *IL6*, *IL6R*, *IL1B*, *RANK* and *RANKL* genes, with fracture.

| **SNV and**  **Model** | **Controls**  **n (%)** | **HFx**^a^  **n (%)** | **OR (95% CI)^b^** | **p-value**^b^ | **OR (95% CI)^c^** | **p-value**^c^ |
| --- | --- | --- | --- | --- | --- | --- |
| ***CYP19***  **rs700518**  Codominant  TT  TC  CC | 109 (53.7)  75 (36.59)  21 (10.24) | 137 (58.30)  86 (36.60)  12 (5.11) | 1.00  0.91 (0.61-1.35)  **0.45 (0.21-0.96)** | 0.652  **0.040** | 0.68 (0.36-1.30)  0.66 (0.22 – 1.96) | 0.251  0.456 |
| Dominant  TC/CC vs TT | 96 (46.83) | 98 (41.70) | 0.81 (0.55-1.18) | 0.28 | 0.68 (0.37-1.24) | 0.211 |
| Recessive  CC vs TT/TC | 21 (10.24) | 12 (5.11) | **0.47 (0.22-0.98)** | **0.045** | 0.76 (0.23-2.19) | 0.614 |
| **rs1062033**  Codominant  CC  CG  GG | 119 (58.05)  70 (34.15)  16 (7.80) | 147 (62.55)  77 (32.77)  11 (4.68) | 1.00  0.89 (0.62-1.42)  0.55 (0.24-1.24) | 0.574  0.154 | 0.69 (0.36-1.31)  0.82 (0.25-2.67) | 0.261  0.746 |
| Dominant  CG/GG vs CC | 86 (41.95) | 88 (37.45) | 0.82 (0.56-1.21) | 0.335 | 0.71 (0.38-1.30) | 0.273 |
| Recessive  GG vs CC/CG | 16 (7.80) | 11 (4.68) | 0.58 (0.26-1.28) | 0.178 | 0.93 (0.29-2.97) | 0.908 |
| **rs4775936**  Codominant  CC  CT  TT | 115 (56.10)  71 (34.63)  19 (9.27) | 140 (59.57)  83 (35.32)  12 (5.11) | 1.00  0.96 (0.64-1.43)  0.51 (0.24-1.11) | 0.843  0.092 | 0.66 (0.34-1.28)  0.81 (0.27-2.38) | 0.225  0.709 |
| Dominant  CT/TT vs CC | 90 (43.90) | 95 (40.43) | 0.86 (0.59-1.26) | 0.461 | 0.69 (0.38-1.27) | 0.241 |
| Recessive  TT vs CC/CT | 19 (9.27) | 12 (5.11) | 0.52 (0.24-1.11) | 0.093 | 0.93 (0.32-2.67) | 0.902 |
| **rs767199**  Codominant  GG  GA  AA | 113 (55.12)  74 (36.10)  18 (8.78) | 141 (60.0)  83 (35.32)  11 (4.68) | 1.00  0.89 (0.60-1.34)  0.48 (0.22-1.07) | 0.601  0.077 | 0.79 (0.41-1.50)  0.78 (0.26-2.37) | 0.477  0.672 |
| Dominant  GA/AA vs GG | 92 (44.88) | 94 (40.0) | 0.81 (0.56-1.19) | 0.302 | 0.79 (0.43-1.44) | 0.445 |
| Recessive  AA vs GG/GA | 18 (8.78) | 11 (4.68) | 0.51 (0.23-1.10) | 0.089 | 0.85 (0.29-2.52) | 0.781 |
| **rs17523880**  Codominant  CC  CA  AA | 179 (87.32)  9 (4.39)  17 (8.29 | 214 (91.06)  0 (0)  21 (8.94) | 1.00  --  1.03 (0.52-2.01) | --  0.924 | --  1.98 (0.75-5.2) | --  0.171 |
| Dominant  CA/AA vs CC | 26 (12.68) | 21 (8.94) | 0.067 (0.36-1.24) | 0.206 | 1.62 (0.64-4.09) | 0.308 |
| Recessive  AA vs CC/CA | 17 (8.29) | 21 (8.94) | 1.08 (0.55-2.11) | 0.811 | 2.03 (0.75-5.44) | 0.158 |
| ***ESR1***  **rs2234693**  Codominant  TT  TC  CC | 97 (47.32)  80 (39.02)  28 (13.66) | 127 (54.04)  86 (36.60)  22 (9.36) | 1.00  0.82 (0.54-1.22)  0.60 (0.32-1.11) | 0.338  0.105 | 1.03 (0.54-1.97)  **0.30 (0.11-0.79)** | 0.924  **0.015** |
| Dominant  TC/CC vs TT | 108 (52.68) | 108 (45.96) | 0.65 (0.52-1.11) | 0.160 | 0.76 (0.42-1.37) | 0.367 |
| Recessive  CC vs TT/TC | 28 (13.66) | 22 (9.36) | 0.62 (0.36-1.18) | 0.159 | **0.30 (0.12-0.75)** | **0.010** |
| **rs2228480**  Codominant  GG  GA  AA | 102 (49.76)  88 (42.93)  15 (7.32) | 108 (45.96)  100 (42.55)  27 (11.49) | 1.00  1.07 (0.72-1.59)  1.7 (085-3.37) | 0.725  0.130 | 0.90 (0.48-1.69)  1.26 (0.43-3.64) | 0.752  0.662 |
| Dominant  GA/AA vs G | 103 (50.24) | 127 (54.04) | 1.16 (0.80-1.69) | 0.426 | 0.96 (0.52-1.74) | 0.896 |
| Recessive  AA vs GG/GA | 15 (7.32) | 27 (11.49) | 1.64 (0.84-3.18) | 0.140 | 1.32 (0.48-3.6) | 0.584 |
| ***IL6***  **rs1800795**  Codominant  GG  GC  CC | 158 (77.07)  44 (21.46)  3 (1.46) | 192 (81.70)  41 (17.45)  2 (0.85) | 1.00  0.76 (0.47-1.26)  0.54 (0.09-3.32) | 0.273  0.514 | 0.96 (0.47-1.97)  0.127 (0.0008-20.23) | 0.922  0.426 |
| Dominant  GC/CC vs GG | 47 (22.93) | 43 (18.30) | 0.75 (0.47-1.19) | 0.231 | 0.92 (04.45-1.86) | 0.821 |
| Recessive  CC vs GG/GC | 3 (1.46) | 2 (0.85) | 0.57 (0.09-3.4) | 0.550 | 0.12 (0.0008-20.46) | 0.428 |
| **rs1800796**  Codominant  GG  GC  CC | 91 (44.39)  81 (39.51)  33 (16.10) | 89 (37.87)  111 (47.23)  35 (14.89) | 1.00  1.40 (0.93-2.10)  1.08 (0.62-1.80) | 0.106  0.776 | 1.32 (0.69-2.53)  2.02 (0.83-4.96) | 0.394  0.121 |
| Dominant  GC/CC vs GG | 114 (55.61) | 146 (62.13) | 1.3 (0.89-1.91) | 0.166 | 1.48 (0.81-2.71) | 0.201 |
| Recessive  CC vs GG/GC | 33 (16.10) | 35 (14.89) | 0.91 (0.54-1.53) | 0.728 | 1.76 (0.76-4.04) | 0.181 |
| ***IL6R***  **rs4845617**  Codominant  GG  GA  AA | 55 (26.83)  99 (48.29)  51 (24.88) | 57 (24.26)  12251.91)  56 23.83) | 1.00  1.18 (0.75-1.87)  1.05 (0.62-1.80) | 0.456  0.831 | 1.04 (0.51-2.11)  1.10 (04.47-2.55) | 0.901  0.819 |
| Dominant | 150 (73.17) | 178 (75.74) | 1.14 (0.74-1.75) | 0.537 | 1.06 (0.54-2.05) | 0.855 |
| Recessive  AA vs GG/GA | 51 (24.88) | 56 (23.83) | 0.94 (0.61-1.46) | 0.798 | 1.07 (0.52-2.19) | 0.848 |
| **rs2228145**  Codominant  CC  CA  AA | 53 (25.85)  114 (55.61)  38 (18.54) | 72 (30.74)  114 (48.51)  49 (20.85) | 1.00  0.73 (0.47-1.14)  0.94 (0.54-1.64) | 0.172  0.853 | 0.82 (0.40-1.67)  0.76 (0.32-1.81) | 0.600  0.550 |
| Dominant  CA/AA vs CC | 152 (74.15) | 163 (69.36) | 0.78 (0.51-1.19) | 0.267 | 0.81 (0.41-1.58) | 0.538 |
| Recessive  AA vs CC/CA | 38 (18.54) | 49 (20.85) | 1.15 (0.72-1.85) | 0.543 | 0.87 (0.42-1.79) | 0.708 |
| ***IL1B***  **rs16944**  Codominant  AA  AG  GG | 69 (33.66)  102 (49.76)  34 (16.59) | 87 (37.02)  117 (49.79)  31 (13.19) | 1.00  0.90 (0.60-1.37)  0.72 (0.40-1.29) | 0.653  0.274 | 0.83 (0.43-1.60)  0.93 (0.38-2.28) | 0.581  0.888 |
| Dominant  AG/GG vs AA | 136 (66.34) | 148 (62.98) | 0.86 (0.58-1.27) | 0.462 | 0.85 (0.46-1.19) | 0.626 |
| Recessive  GG vs AA/AG | 34 (16.59) | 31 (13.19) | 0.76 (0.45-1.29) | 0.318 | 1.04 (0.46-2.33) | 0.918 |
| ***RANK***  **rs3018362**  Codominant  GG  GA  AA | 67 (32.68)  105 (51.22)  33 (16.10) | 65 (27.66)  121 (51.49)  49 (20.85) | 1.00  1.18 (0.77-1.82)  1.53 (0.87-2.67) | 0.433  0.135 | 1.4 (0.70-2.80)  **2.4 (1.01-6.06)** | 0.335  **0.047** |
| Dominant  GA/AA vs GG | 138 (67.32) | 170 (72.34) | 1.26 (0.84-1.91) | 0.252 | 1.63 (0.84-3.14) | 0.143 |
| Recessive  AA vs AA/GA | 33 (16.10) | 49 (20.85) | 1.37 (0.84-2.23) | 0.203 | 1.99 (0.92-4.33) | 0.080 |
| ***RANKL***  **rs12585014**  Codominant  GG  GA  AA | 81 (39.51)  87 (42.44)  37 (18.05) | 89 (37.87)  106 (45.11)  40 (17.02) | 1.00  1.10 (0.73-1.67)  0.98 (0.57-1.68) | 0.624  0.953 | 1.09 (0.57-2.11)  1.29 (0.56-2.99) | 0.779  0.542 |
| Dominant  GA/AA vs GG | 124 (60.49) | 146 (62.13) | 1.07 (0.72-1.57) | 0.725 | 1.15 (0.63-2.11) | 0.641 |
| Recessive  AA vs GG/GA | 37 (18.05) | 40 (17.02) | 0.93 (0.56-1.52) | 0.77 | 1.23 (0.57-2.65) | 0.587 |

a: hip fracture; b: unadjusted data; c: adjusted by age, BMI, estrogen intake and diabetes; significant values are shown in bold.
